# Bioinformatics analysis of potential common pathogenic mechanism for carotid atherosclerosis and Parkinson’s disease

**DOI:** 10.3389/fnagi.2023.1202952

**Published:** 2023-08-15

**Authors:** Quan Wang, Qun Xue

**Affiliations:** Department of Neurology, The First Affiliated Hospital of Soochow University, Suzhou, China

**Keywords:** atherosclerosis, Parkinson’s disease, bioinformatics analysis, hub genes, immune cells infiltration

## Abstract

**Background:**

Cerebrovascular disease (CVD) related to atherosclerosis and Parkinson’s disease (PD) are two prevalent neurological disorders. They share common risk factors and frequently occur together. The aim of this study is to investigate the association between atherosclerosis and PD using genetic databases to gain a comprehensive understanding of underlying biological mechanisms.

**Methods:**

The gene expression profiles of atherosclerosis (GSE28829 and GSE100927) and PD (GSE7621 and GSE49036) were downloaded from the Gene Expression Omnibus (GEO) database. After identifying the common differentially expressed genes (DEGs) for these two disorders, we constructed protein-protein interaction (PPI) networks and functional modules, and further identified hub genes using Least Absolute Shrinkage and Selection Operator (LASSO) regression. The diagnostic effectiveness of these hub genes was evaluated using Receiver Operator Characteristic Curve (ROC) analysis. Furthermore, we used single sample gene set enrichment analysis (ssGSEA) to analyze immune cell infiltration and explored the association of the identified hub genes with infiltrating immune cells through Spearman’s rank correlation analysis in R software.

**Results:**

A total of 50 shared DEGs, with 36 up-regulated and 14 down-regulated genes, were identified through the intersection of DEGs of atherosclerosis and PD. Using LASSO regression, we identified six hub genes, namely C1QB, CD53, LY96, P2RX7, C3, and TNFSF13B, in the lambda.min model, and CD14, C1QB, CD53, P2RX7, C3, and TNFSF13B in the lambda.1se model. ROC analysis confirmed that both models had good diagnostic efficiency for atherosclerosis datasets GSE28829 (lambda.min AUC = 0.99, lambda.1se AUC = 0.986) and GSE100927 (lambda.min AUC = 0.922, lambda.1se AUC = 0.933), as well as for PD datasets GSE7621 (lambda.min AUC = 0.924, lambda.1se AUC = 0.944) and GSE49036 (lambda.min AUC = 0.894, lambda.1se AUC = 0.881). Furthermore, we found that activated B cells, effector memory CD8 + T cells, and macrophages were the shared correlated types of immune cells in both atherosclerosis and PD.

**Conclusion:**

This study provided new sights into shared molecular mechanisms between these two disorders. These common hub genes and infiltrating immune cells offer promising clues for further experimental studies to explore the common pathogenesis of these disorders.

## 1. Introduction

Cerebrovascular disease (CVD) and Parkinson’s disease (PD) are both age-related conditions that contribute significantly to the global burden of neurological disorders, which are the leading causes of mortality and morbidity (Collaborators GBDP, 2018, 2019a,b). Cerebrovascular accidents are the second largest cause of mortality globally, with over 80 million survivors in 2016 experiencing varying degrees of decline in their quality of life (Collaborators GBDP, 2019a). Ischemic cerebrovascular events accounted for over 80% of all cerebrovascular accidents, with atherosclerosis being the most common cause (Collaborators GBDP, 2019b; Song et al., 2020). Notably, moderate carotid artery stenosis caused by atherosclerosis affected 4.8% of men and 2.2% of women under 70 years of age. This condition’s prevalence escalated significantly among individuals aged 70 and older, reaching 12.5% in men and 6.9% in women within this age group (de Weerd et al., 2009). PD is the second most prevalent neurodegenerative disorder, affecting 6.1 million individuals worldwide in 2016, and its prevalence is expected to continue rising as life expectancy increases (Collaborators GBDP, 2018). PD patients experience movement disorders due to progressive impairments in the nigrostriatal system, which can significantly impact their ability to care for themselves (Bloem et al., 2021). Therefore, extensive research into these diseases, along with the development of effective preventative methods, is critical in reducing the global burden of neurological disorders.

Parkinson’s disease and CVD share some risk factors such as older age, male gender, and diabetes mellitus. However, moderate coffee consumption has been associated with a lower risk of both PD and CVD (Potashkin et al., 2020). The underlying causes of PD are not yet fully understood, but research has identified genetic and environmental risk factors (Bloem et al., 2021). Pathogenic mutations in certain genes have been linked to an increased risk of developing sporadic PD or familial parkinsonism (Blauwendraat et al., 2020). Investigations of PD-related genes have revealed that the clearance of damaged mitochondria, which contributes to oxidative stress and inflammatory processes, is disrupted in some cases of familial parkinsonism (Sliter et al., 2018). Furthermore, immunological responses to α-synuclein have been found in a significant percentage of patients with sporadic PD (Sulzer et al., 2017), suggesting a possible role of central nervous system inflammation in this disease. Similarly, inflammation mediated by various cardiovascular risk factors has been identified as a crucial factor in the formation of atherosclerotic plaques (Soehnlein and Libby, 2021).

In clinical practice, the co-occurrence of PD and CVD is relatively common. Research has demonstrated that people with PD are nearly twice as likely to have atherosclerosis as those without PD (Alves et al., 2021). Additionally, postmortem studies have also suggested that vascular pathology may be a contributing factor in the underlying pathogenesis of PD (Hong et al., 2018). The clinical concept of vascular parkinsonism can arise from strategic infarcts in the basal ganglia or diffuse periventricular white matter lesions due to cerebral small vessel disease (Narasimhan et al., 2022). Beyond the motor symptoms, studies have indicated that carotid atherosclerosis contributes to microvascular injury, leading to worsened cognitive dysfunction in PD, significantly impacting the daily quality of life for patients (Kim et al., 2012, 2014). Understanding and addressing these aspects of PD and CVD co-occurrence are crucial for comprehensive patient care and management. Since inflammation plays a role in the development of both atherosclerosis and PD, it raises interesting questions about the shared inflammatory pathways involved in two diseases. Accumulating evidence indicates that both conditions are associated with elevated levels of C-reactive protein, which activates the complement system (Song et al., 2011; Badimon et al., 2018). Moreover, the NLRP3 (NLR family pyrin domain containing 3) inflammasome contributes to the progression of atherosclerosis, and inhibiting its assembly in both familial and sporadic PD models reduces dopaminergic neurodegeneration (Duewell et al., 2010; Panicker et al., 2022). Therefore, targeting common inflammatory pathways may offer a promising therapeutic approach for managing patients with these comorbidities.

Given the significant prevalence and health burden imposed by PD and CVD, further understanding of their shared risk factors could provide valuable insight into underlying pathophysiological mechanisms and novel targets for prevention and treatment. Therefore, we conducted a study to investigate the association between atherosclerosis and PD using genetic databases. Our aim was to integrate and analyze gene data related to the pathogenesis of atherosclerosis and PD to gain new insights into the biological mechanisms of these two diseases, and to facilitate the development of preventative measures for both.

## 2. Materials and methods

### 2.1. Acquisition and preparation of datasets

We acquired datasets of array-based expression profiling from the Gene Expression Omnibus (GEO) database,^1^ a public functional genomics database containing a vast amount of expression microarray data. To identify relevant gene expression datasets from human tissues, we conducted a search using the keywords “Parkinson’s disease” and “atherosclerosis.” Subsequently, we included the PD datasets GSE7621 and GSE49036, as well as the atherosclerosis datasets GSE28829 and GSE100927 in our analysis after ensuring their suitability for our study.

### 2.2. Analysis of differentially expressed genes (DEGs) and identification of shared DEGs

In this study, we utilized the tinyarray R package (version 2.2.9) to perform a differential analysis of count data based on expression sets and group information. DEGs were defined as genes with an adjusted *P*-value less than 0.05 and an absolute log2 fold change (log2FC) greater than 0.5. To visualize the DEGs, including hierarchical clustering heatmaps, volcano plots, and principal component analysis (PCA) plots, we used the ggplot2 R package (version 3.4.1). The shared DEGs between the GSE49036 and GSE28829 datasets were illustrated using the VennDiagram R package (version 1.7.3).

### 2.3. Construction of the protein-protein interaction (PPI) network of shared DEGs

To obtain the interaction relationships between shared DEGs, we used the Search Tool for the Retrieval of Interacting Genes (STRING)^2^ database. The PPI network of these shared DEGs was constructed with a minimum required interaction score of 0.4. We visualized the PPI using Cytoscape software (version 3.9.1) and identified core functional modules from the PPI using the molecular complex detection (MCODE) plug-in (version 2.0.2). The parameters used in this study were as follows: degree cutoff = 2, node density cutoff = 0.1, node score cutoff = 0.2, K-score = 2, and max depth = 100.

### 2.4. Identification of hub genes using least absolute shrinkage and selection operator (LASSO) regression

The GSE28829 dataset was used as the training dataset to construct a diagnostic model. The hub genes obtained from the shared DEGs of the GSE49036 and GSE28829 datasets were further narrowed down to six genes using LASSO regression analysis through the GLMNET R package (version 4.1-7). The significant differential expressions of these hub genes between the atherosclerosis group and the control group in the training dataset were identified using the Wilcoxon test. Additionally, ROC curves were constructed using the ROCR R package (version 1.0-11), and the AUC was calculated to assess the diagnostic effectiveness of these hub genes in the training dataset.

To validate the diagnostic effectiveness of the six hub genes identified from the training dataset GSE28829, we further tested their expression levels in the verification datasets GSE7621, GSE49036, and GSE100927. The differential expression of hub genes between the disease group and control group was determined by Wilcoxon test. Furthermore, ROC curves were constructed using the ROCR R package (version 1.0-11) and the AUC was calculated to evaluate the diagnostic effectiveness of these hub genes in the verification datasets.

### 2.5. Enrichment analysis of DEGs

Enrichment analysis of DEGs is a widely used approach to gain insights into the biological functions and pathways of target genes (Reimand et al., 2019). In this study, we performed Gene Ontology (GO) and Kyoto Encyclopedia of Genes and Genomes (KEGG) enrichment analyses to identify potential molecular pathways that are involved in atherosclerosis and PD. We conducted enrichment analyses for both individual DEGs and shared DEGs obtained from GSE49036 and GSE28829 datasets. A threshold of adjusted *P*-value < 0.05 was used to determine statistical significance.

### 2.6. Correlation analysis of immune cell infiltration

To determine the types of infiltrating immune cells present in atherosclerosis and PD based on expression profiles obtained from pathological tissue samples, we conducted immune cell infiltration analysis using single sample gene set enrichment analysis (ssGSEA) in the GSE49036 and GSE28829 datasets. The GSVA R package (version 1.46.0) was used to conduct the analysis, using a gene set consisting of 782 reference cell markers. The abundance of 28 types of immune cells was predicted based on the expression levels of these markers, including activated B cells (Ba), immature B cells (Bi), memory B cells (Bm), activated CD4 + T cells (CD4 + Ta), activated CD8 + T cells (CD8 + Ta), central memory CD4 + T cells (CD4 + T cm), central memory CD8 + T cells (CD8 + T cm), effector memory CD4 + T cells (CD4 + Tem), effector memory CD8 + T cells (CD8 + Tem), gamma delta T cells (γδT), regulatory T cells (Treg), follicular helper T cells (Tfh), type-1 T helper cells (Th1), type-2 T helper cells (Th2), type-17 T helper cells (Th17), natural killer T cells (NKT), natural killer cells (NK), CD56 bright natural killer cells (CD56 + NK), CD56 dim natural killer cells (CD56-NK), activated dendritic cells (DCa), immature dendritic cells (DCi), plasmacytoid dendritic cells (DCp), neutrophils, eosinophils, monocytes, macrophages, mast cells, and myeloid-derived suppressor cells (MDSCs).

## 3. Results

### 3.1. Gene expression omnibus (GEO) dataset information

This study employed four datasets acquired from the GEO database, comprising of two atherosclerosis datasets (GSE28829 and GSE100927) and two PD datasets (GSE7621 and GSE49036). A detailed summary of each dataset is presented in Table 1.

**TABLE 1:** Detailed information of GEO datasets.

ID	GSE number	Disease	Platform	Samples
1	GSE7621	Parkinson’s disease	GPL570	16 patients and 9 controls
2	GSE49036	Parkinson’s disease	GPL570	20 patients and 8 controls
3	GSE28829	Atherosclerosis	GPL570	16 patients and 13 controls
4	GSE100927	Atherosclerosis	GPL17077	69 patients and 35 controls

### 3.2. Identification of DEGs and shared DEGs between atherosclerosis and PD datasets

**FIGURE 1:**
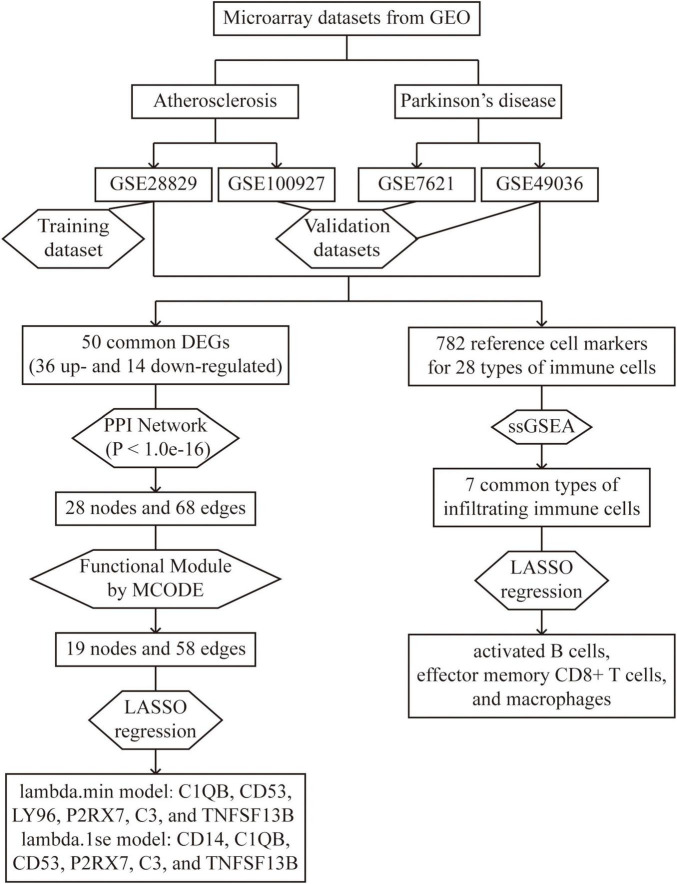
Overall flowchart of this study: two atherosclerosis datasets and two Parkinson’s datasets were analyzed in this study to identify potential hub genes and types of infiltrating immune cells.

**FIGURE 2:**
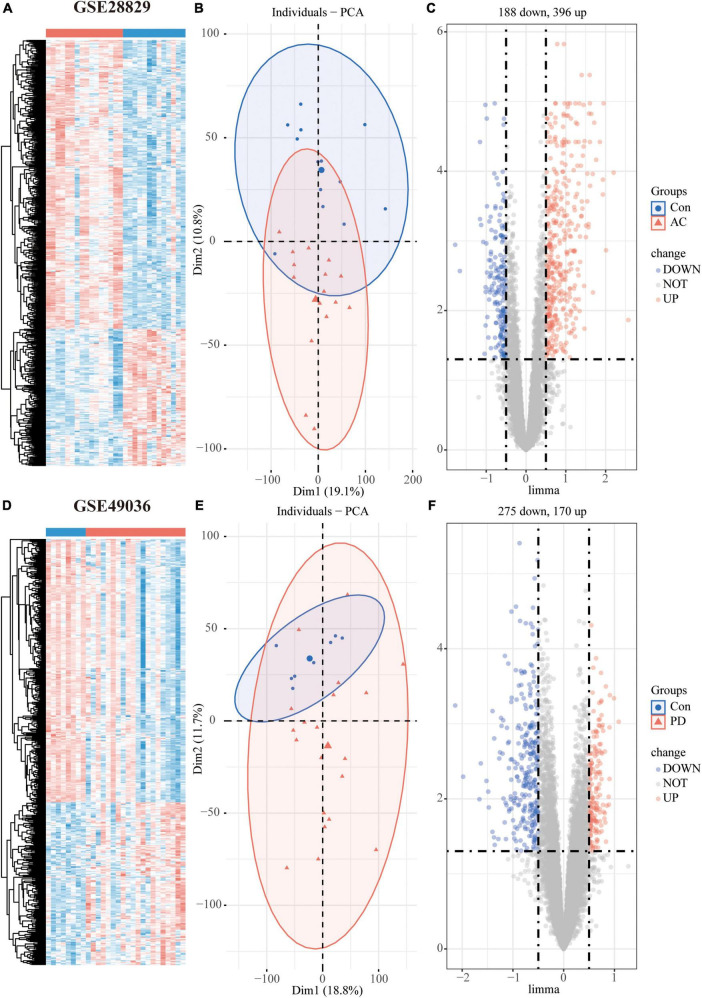
Differentially expressed genes (DEGs) analysis of individual datasets: Hierarchical clustering heatmap **(A)**, PCA plot **(B)**, and volcano plot **(C)** of atherosclerosis dataset GSE28829; Hierarchical clustering heatmap **(D)**, PCA plot **(E)**, and volcano plot **(F)** of PD dataset GSE49036.

The study’s overall flowchart is presented in Figure 1. In the atherosclerosis datasets, we found 584 DEGs (396 up-regulated and 188 down-regulated genes) in GSE28829 (Figures 2A–C) and 2128 DEGs (1192 up-regulated and 936 down-regulated genes) in GSE100927 (Supplementary Figures 1A–C) compared to control samples. In the PD datasets, we identified 445 DEGs (170 up-regulated and 275 down-regulated genes) in GSE49036 (Figures 2D–F) and 809 DEGs (403 up-regulated and 406 down-regulated genes) in GSE7621 (Supplementary Figures 1D–F) compared to control samples.

To determine the common DEGs in atherosclerosis and PD datasets, we took the intersection of up-regulated and down-regulated genes from GSE28829 and GSE49036. This analysis identified 50 shared DEGs, consisting of 36 up-regulated and 14 down-regulated genes, as shown in the Venn diagram in Supplementary Figure 2A.

### 3.3. PPI network analysis and functional module analysis

**FIGURE 3:**
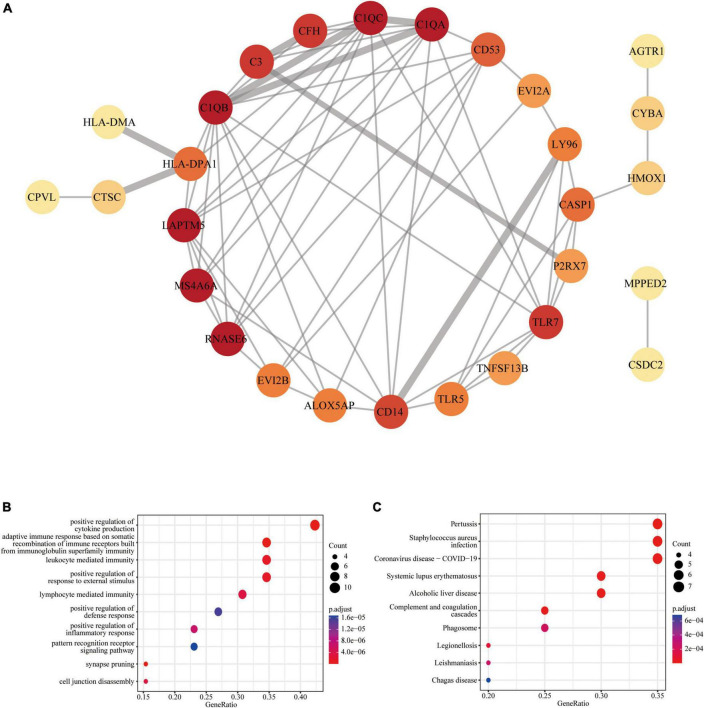
Protein-protein interaction (PPI) network of common DEGs: **(A)** PPI network with 28 nodes and 68 edges; **(B)** the GO enrichment result of these 28 common DEGs; **(C)** the KEGG enrichment result of these 28 common DEGs.

The PPI network analysis resulted in a network with 28 nodes and 68 edges, with the PPI enrichment *P*-value lower than 1.0e-16 (Figure 3A). We used Cytoscape software to visualize the PPI network, where we color-coded the genes based on their connectivity. Genes that were highly connected appeared in redder color. GO enrichment analysis of these genes in PPI network showed that the top five associated biological processes were positive regulation of cytokine production, adaptive immune response based on somatic recombination of immune receptors built from immunoglobulin superfamily domains, leukocyte mediated immunity, positive regulation of response to external stimulus, and lymphocyte mediated immunity (Figure 3B). KEGG enrichment analysis indicated that the top five terms associated with these genes were pertussis, Staphylococcus aureus infection, coronavirus disease—COVID-19, systemic lupus erythematosus, and alcoholic liver disease (Figure 3C).

**FIGURE 4:**
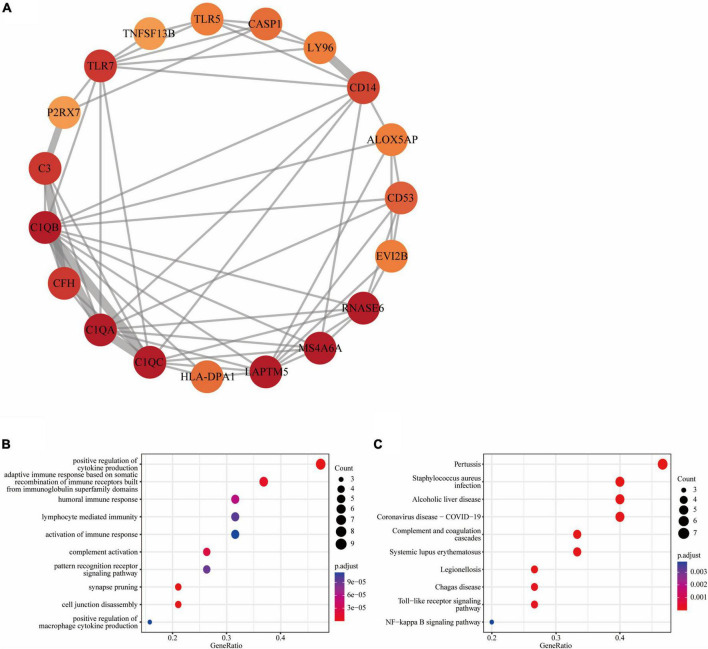
Functional module of common DEGs: **(A)** PPI network of the key functional module with 19 nodes and 58 edges; **(B)** the GO enrichment result of these 19 common DEGs; **(C)** the KEGG enrichment result of these 19 common DEGs.

We further obtained a key functional module that included 19 DEGs by applying the MCODE plug-in of Cytoscape (Figure 4A). GO enrichment analysis of these genes in key functional module revealed that the top five associated biological processes were positive regulation of cytokine production, adaptive immune response based on somatic recombination of immune receptors built from immunoglobulin superfamily domains, humoral immune response, lymphocyte mediated immunity, and activation of immune response (Figure 4B). KEGG enrichment analysis highlighted the top five terms associated with these genes were pertussis, Staphylococcus aureus infection, alcoholic liver disease, coronavirus disease—COVID-19, and complement and coagulation cascades (Figure 4C).

### 3.4. Identification and validation of hub genes

**FIGURE 5:**
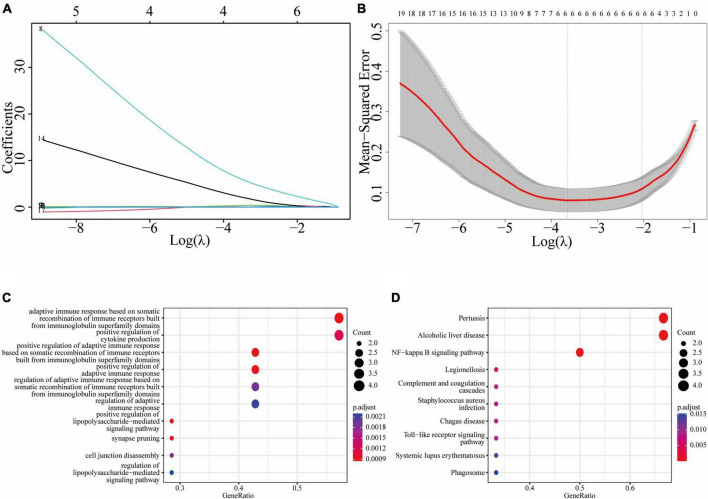
Least absolute shrinkage and selection operator (LASSO) regression model based on 19 common DEGs: **(A)** LASSO coefficient profiles of the 19 prognostic DEGs; **(B)** cross-validation to select the optimal tuning parameter (λ); **(C)** the GO enrichment result of the common hub genes; **(D)** the KEGG enrichment result of the common hub genes.

We used the atherosclerosis dataset GSE28829 as our training dataset to identify the top hub genes using LASSO regression. From the 19 shared DEGs in the key functional module, we narrowed down to six hub genes, namely C1QB, CD53, LY96, P2RX7, C3, and TNFSF13B in the lambda.min model, and CD14, C1QB, CD53, P2RX7, C3, and TNFSF13B in the lambda.1se model (Figures 5A, B). GO enrichment analysis of these hub genes revealed that they were mainly associated with adaptive immune response, including adaptive immune response based on somatic recombination of immune receptors built from immunoglobulin superfamily domains, cytokine production, lipopolysaccharide-mediated signaling pathway, synapse pruning, and cell junction disassembly (Figure 5C). KEGG enrichment analysis indicated that these genes were mainly associated with pertussis, alcoholic liver disease, and NF-κB signaling pathway, legionellosis, complement and coagulation cascades, staphylococcus aureus infection, Chagas disease, toll-like receptor signaling pathway, systemic lupus erythematosus, and phagosome (Figure 5D).

**FIGURE 6:**
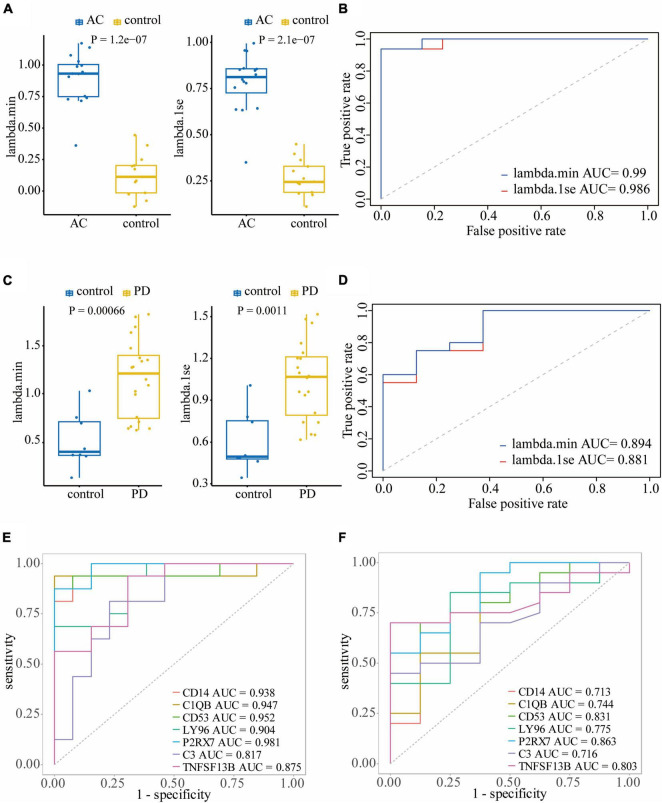
Validating the diagnostic efficacy of hub genes: Wilcoxon test **(A)** and ROC curve **(B)** of the diagnostic efficacy of hub genes in atherosclerosis dataset GSE28829; Wilcoxon test **(C)** and ROC curve **(D)** of the diagnostic efficacy of hub genes in PD dataset GSE49036. **(E)** The ROC curves of the diagnostic efficacy of individual hub genes in GSE28829 dataset; **(F)** the ROC curves of the diagnostic efficacy of individual hub genes in GSE49036 dataset.

To assess the diagnostic performance of LASSO regression models in differentiating patients from controls, we conducted Wilcoxon tests and generated ROC curves. In the training dataset GSE28829, both the lambda.min model (*P* = 1.2e-7) and lambda.1se model (*P* = 2.1e-7) showed a statistically significant difference between atherosclerosis and control samples in the Wilcoxon test (Figure 6A). These models exhibited excellent diagnostic efficiency, with the lambda.min model having an AUC of 0.99 and the lambda.1se model having an AUC of 0.986 (Figure 6B). Similarly, in the atherosclerosis dataset GSE100927, both models showed a statistically significant difference between atherosclerosis and control samples in the Wilcoxon test, with the lambda.min model having a *P*-value of 2.5e-12 and the lambda.1se model having a *P*-value of 6.2e-13 (Supplementary Figure 3A). Both models also demonstrated good diagnostic efficiency, with the lambda.min model having an AUC of 0.922 and the lambda.1se model having an AUC of 0.933 (Supplementary Figure 3B).

In the PD dataset GSE49036, which was used to identify the common DEGs with the training dataset, both models showed a statistically significant difference between atherosclerosis and control samples in the Wilcoxon test, with the lambda.min model having a *P*-value of 0.00066 and the lambda.1se model having a *P*-value of 0.0011 (Figure 6C). Both models demonstrated good diagnostic efficiency, with the lambda.min model having an AUC of 0.894 and the lambda.1se model having an AUC of 0.88 (Figure 6D). Likewise, in the PD dataset GSE7621, both models exhibited a statistically significant difference between atherosclerosis and control samples in the Wilcoxon test, with the lambda.min model having a *P*-value of 0.00019 and the lambda.1se model having a *P*-value of 6.6e-05 (Supplementary Figure 3C). Both models demonstrated good diagnostic efficiency, with the lambda.min model having an AUC of 0.924 and the lambda.1se model having an AUC of 0.944 (Supplementary Figure 3D).

Furthermore, we assessed the diagnostic efficacy of individual hub genes. In the atherosclerosis dataset GSE28829, CD14 (AUC = 0.938), C1QB (AUC = 0.947), CD53 (AUC = 0.952), LY96 (AUC = 0.904), P2RX7 (AUC = 0.981), C3 (AUC = 0.817), and TNFSF13B (AUC = 0.875) demonstrated favorable diagnostic efficiency for differentiating patients with atherosclerosis from controls (Figure 6E). In the PD dataset GSE49036, CD14 (AUC = 0.713), C1QB (AUC = 0.744), CD53 (AUC = 0.831), LY96 (AUC = 0.775), P2RX7 (AUC = 0.863), C3 (AUC = 0.716), and TNFSF13B (AUC = 0.803) also exhibited good diagnostic efficiency in differentiating patients with PD from controls (Figure 6F).

### 3.5. Correlation between hub genes and immune cell infiltration

**FIGURE 7:**
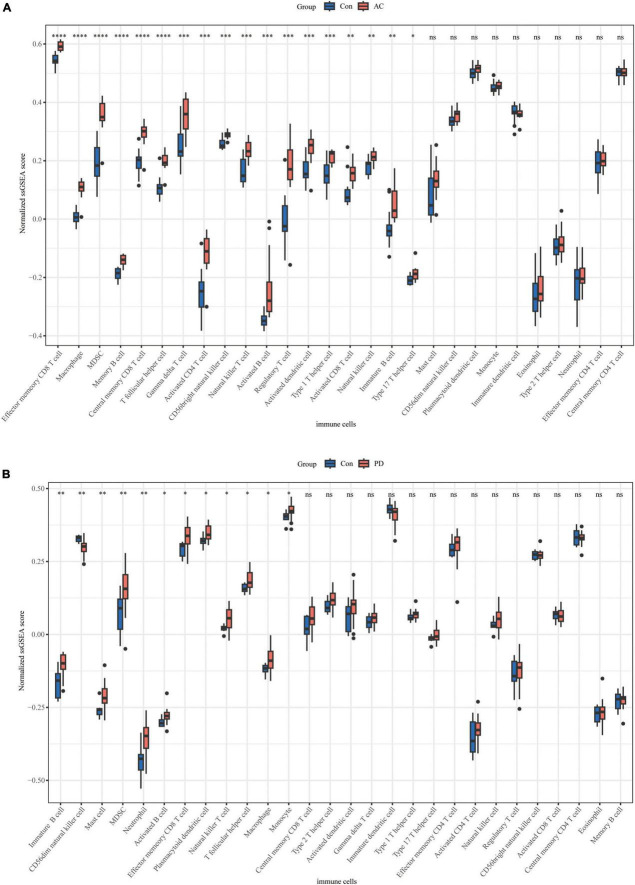
Immune cell infiltration of individual datasets: **(A)** boxplot of normalized ssGSEA scores of 28 types of immune cells in atherosclerosis dataset GSE28829; **(B)** boxplot of normalized ssGSEA scores of 28 types of immune cells in PD dataset GSE49036. *P < 0.05, ^**^P < 0.01, ^***^P < 0.001, ^****^P < 0.0001.

**FIGURE 8:**
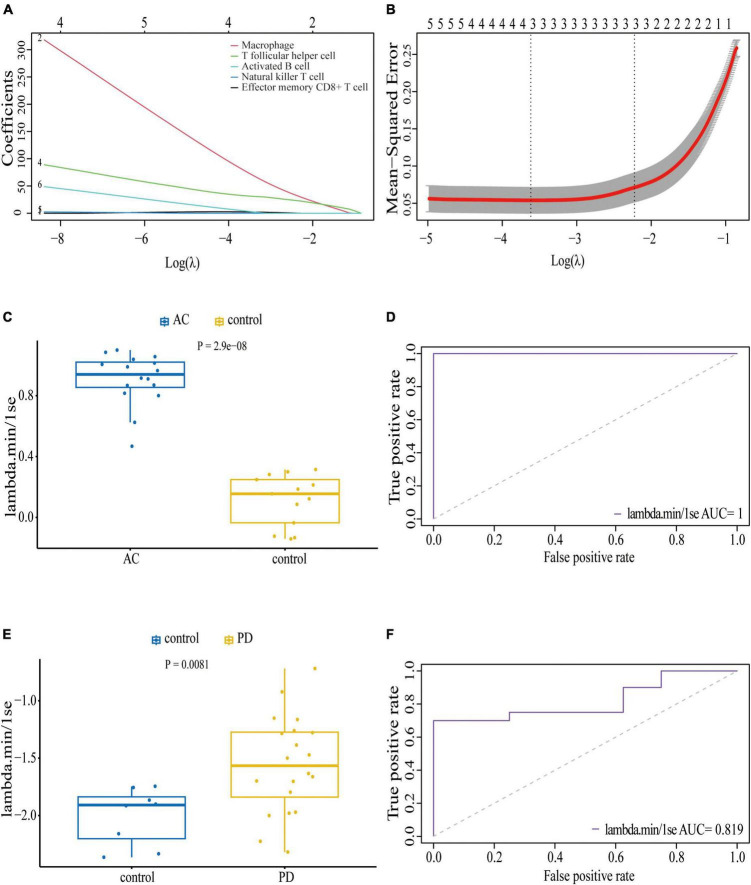
Least absolute shrinkage and selection operator (LASSO) regression model based on seven common infiltrating immune cells: **(A)** LASSO coefficient profiles of the seven prognostic infiltrating immune cells; **(B)** cross-validation to select the optimal tuning parameter (λ); Wilcoxon test **(C)** and ROC curve **(D)** of the diagnostic efficacy of key common infiltrating immune cells in atherosclerosis dataset GSE28829; Wilcoxon test **(E)** and ROC curve **(F)** of the diagnostic efficacy of key common infiltrating immune cells in PD dataset GSE49036.

We conducted an immune cell infiltration analysis in the GSE49036 and GSE28829 datasets using ssGSEA, and the results are presented in box plots. The analysis revealed that 18 types of immune cells, including CD8 + Tem, macrophages, MDSCs, Bm, CD8 + T cm, Tfh, γδT, CD4 + Ta, CD56 + NK, NKT, Ba, Treg, DCa, Th1, CD8 + Ta, NK, Bi, and Th17, were different between patients with atherosclerosis and controls in the GSE28829 dataset (Figure 7A). In the PD dataset GSE49036, 12 types of immune cells, including Bi, CD56-NK, mast cells, MDSCs, neutrophils, Ba, CD8 + Tem, DCp, NKT, Tfh, macrophages, and monocytes, were found to be different between patients with PD and controls (Figure 7B). The common types of immune cells that were correlated with both atherosclerosis and PD were CD8 + Tem, macrophages, MDSCs, Tfh, NKT, Ba, and Bi, as shown in Supplementary Figure 2B. Furthermore, we used the GSE28829 dataset as the training dataset and applied LASSO regression to narrow down the shared types of infiltrating immune cells. In both the lambda.min and lambda.1se models, Ba, CD8 + Tem, and macrophages were identified as the key types of infiltrating immune cells (Figures 8A, B).

We also assessed the diagnostic efficacy of these LASSO regression models by conducting Wilcoxon tests and generating ROC curves. In the atherosclerosis dataset GSE28829, which was used as the training dataset, these infiltrating immune cells exhibited a statistically significant difference between patient and control samples in the Wilcoxon test (Figure 8C). The model demonstrated excellent predictive efficiency (AUC = 1) for differentiating patients with atherosclerosis from controls (Figure 8D). Similarly, in the PD dataset GSE49036, which was used to identify the common types of infiltrating immune cells with the training dataset, these types of infiltrating immune cells also showed a statistically significant difference between patient and control samples in the Wilcoxon test (Figure 8E). The model exhibited good diagnostic efficiency (AUC = 0.819) for differentiating patients with PD from controls (Figure 8F).

**FIGURE 9:**
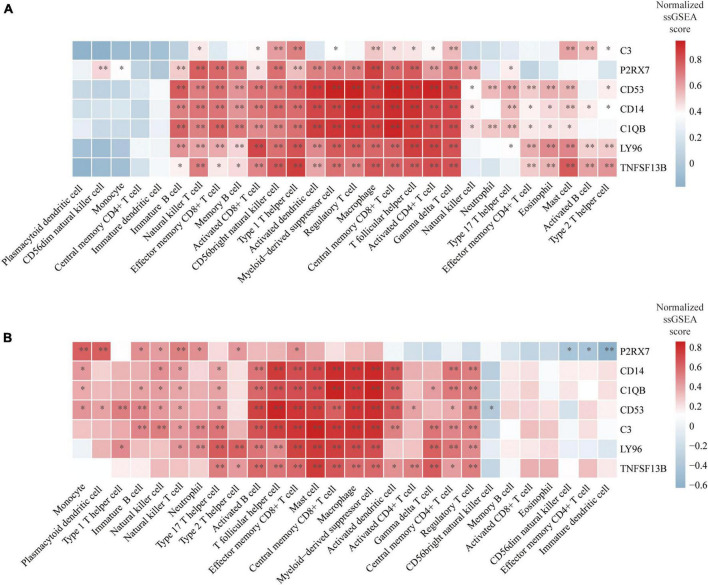
Correlation between hub genes and immune cells: **(A)** Heatmap of the correlation between hub genes and immune cells in atherosclerosis dataset GSE28829; **(B)** heatmap of the correlation between hub genes and immune cells in PD dataset GSE49036. *P < 0.05, ^**^P < 0.01.

We finally evaluated the correlation between hub genes and infiltrating immune cells identified in this study. The correlation results were visualized using heatmaps. The redder or bluer the color of the gene in the immune cell column, the higher the positive or negative association of the gene with this type of immune cell. In the reference cell markers of immune cells used in this study, CD14 is considered as a marker of MDSCs, C1QB is considered as a marker of DCa, and CD53 is considered as a marker of Th1. They were found to be significantly associated with multiple types of immune cells in both atherosclerosis and PD patients. Additionally, the remaining four hub genes were also significantly correlated with multiple types of immune cells (Figures 9A, B).

## 4. Discussion

The objective of this study was to identify the most relevant genes to both atherosclerosis and PD. Initially, 50 shared differentially expressed genes (DEGs) were identified from both conditions and used to construct a protein-protein interaction (PPI) network. From this network, functional modules were extracted to select 19 hub genes. These hub genes included ALOX5AP, CD53, LAPTM5, CD14, C1QB, EVI2B, C1QA, C3, RNASE6, CFH, C1QC, TLR7, MS4A6A, HLA-DPA1, P2RX7, CASP1, LY96, TLR5, and TNFSF13B. To refine the selection of genes, a LASSO regression was carried out. This led to the identification of six genes in the lambda.min model and lambda.1se model, respectively. Among these genes, five were highlighted as being shared between both conditions and therefore the most relevant to both atherosclerosis and PD. These shared genes were C1QB, C3, CD53, P2RX7, and TNFSF13B.

Complement C1q subcomponent subunit B (C1QB) and C3 (complement C3) are important components of the complement system. C1QB is a subunit of the C1q protein, which is the initiator of the classical complement pathway, while C3 plays a central role in all three complement activation pathways (classical, alternative, and lectin pathways) (Walport, 2001a,b). There is a well-established association between the complement system and the development of atherosclerosis (Kiss and Binder, 2022). Specifically, several complement components, including C1q, C3, C4, and C9, have been found to be present in higher concentrations in the plaques and surrounding areas of the intima compared to normal intima (Hollander et al., 1979; Vlaicu et al., 1985). C3, in particular, has been shown to have protective effects against the development of atherosclerotic plaques. Studies using atherosclerotic model mice have found that the absence of C3 leads to elevated triglyceride levels, a proatherogenic lipid profile, and the development of unstable atherosclerotic lesions with a high content of macrophages and low collagen content (Buono et al., 2002; Persson et al., 2004). Other complement components involved in initiating the cascade have generally been found to have protective effects by enhancing the clearance of apoptotic cells, with C1q being one of the most extensively studied molecules in this regard. Studies have demonstrated that the absence of C1q in model mice exacerbated the development of aortic root lesions (Bhatia et al., 2007; Lewis et al., 2009). On the other hand, the complement system has also gained significant attention as a potential regulator of inflammatory responses in PD. Studies have demonstrated that complement components, including C1q, C3, C4, and C9, are present in Lewy bodies and oligodendroglia in the substantia nigra in both sporadic and familial PD, with changes in complement factors also found in their blood (Yamada et al., 1992; McGeer and McGeer, 2004; Goldknopf et al., 2006; Loeffler et al., 2006; Depboylu et al., 2011a). Additionally, studies have found that α-synuclein, a protein associated with neurodegeneration in PD, activates the classical complement cascade, leading to cell death (Gregersen et al., 2021; Ma et al., 2021). However, studies investigating the therapeutic potential of the complement system in PD have yielded mixed results. One study using the MPTP toxin-induced mouse model found that the absence of C3 did not provide protection against dopaminergic neuron loss (Liang et al., 2007). Another study observed an increase in C1q in critical brain areas, but C1q deficiency did not offer disease protection (Depboylu et al., 2011b). However, recent research has implicated knocking out CR3 in mice protected against dopaminergic neuron loss and motor impairment, suggesting that complement opsonization and CR3 engagement play a role in the disease process (Hou et al., 2018). Taken together, while the potential therapeutic benefits of regulating the complement system in atherosclerosis and PD have been explored in model mice, the lack of clinical data and incomplete understanding of the complement system’s role in these diseases highlight the need for additional research to identify potential therapeutic targets.

Tumor necrosis factor ligand superfamily member 13B (TNFSF13B) is a protein also known as BAFF (B-cell activating factor) or BLyS (B-lymphocyte stimulator). It is a cytokine that is primarily produced by immune cells take part into the B cell survival, maturation, and activity (Vincent et al., 2013). The role of TNFSF13B in the development of atherosclerosis is still under debate. Studies in mice have shown that depleting BAFF-R, the receptor of TNFSF13B, can reduce B2 cells and then decrease arterial inflammation, leading to a limitation of atherosclerotic plaque development (Kyaw et al., 2012; Sage et al., 2012). However, the increased levels of soluble TNFSF13B in BAFF-R deficient mice indicate that TNFSF13B itself, other than BAFF-R, could be an atheroprotective factor (Ponnuswamy et al., 2017). Additional research has proposed a potential atheroprotective role of TNFSF13B. Overexpression of TNFSF13B can activate the BAFF-binding receptor TACI, promoting the production of anti-oxLDL IgM and reducing atherosclerosis (Jackson et al., 2016). Furthermore, specific deletion of TACI in myeloid cells has resulted in increased atherosclerosis (Tsiantoulas et al., 2018). Taken together, the TNFSF13B seems to have diverse effect on the development of atherosclerosis via different receptors. A study has shown that the TNFSF13B antibody improved atherosclerosis lesions in mice with low plasma cholesterol levels but worsened the lesions in mice with high cholesterol levels, further indicating the diverse effect of TNFSF13B (Saidoune et al., 2021). Although B cells have not been detected in the cerebrospinal fluid of patients with PD, studies have shown that the levels of B cells in peripheral blood decrease in PD patients (Stevens et al., 2012; Schröder et al., 2018). However, there is currently no direct evidence demonstrating the involvement of TNFSF13B in PD progression. It has been shown that impaired signaling through the BAFF-R receptor results in accelerated disease progression in an animal model of inherited amyotrophic lateral sclerosis, another common neurodegenerative disease. Further study has shown that deficiency of BAFF-R or genetic depletion of B cells does not affect the disease progression, indicating that TNFSF13B-mediated signals on neurons, rather than on B cells, support neural cell survival (Tada et al., 2013). Additionally, APRIL (also known as TNFSF13), another member of the TNF superfamily, has been shown to enhance axon growth during the development of nigrostriatal axons in the striatum (McWilliams et al., 2017). Collectively, these findings suggest that TNFSF13B may have a neuroprotective effect in neurodegenerative diseases, but further investigations are required to confirm this hypothesis. Overall, further research is required to gain a comprehensive understanding of the complex role of TNFSF13B in the pathogenesis of atherosclerosis and PD.

CD53 is a member of the tetraspanin family and is predominantly expressed on B cells and myeloid cells within the immune compartment. Despite its potential importance in immune function, there has been limited research conducted on the specific role of CD53 (Dunlock, 2020). Notably, its involvement in the development of atherosclerosis and PD also has been poorly investigated.

P2X purinoceptor 7 (P2RX7) is an ATP receptor that acts as a ligand-gated ion channel and is predominantly expressed in the nervous and immune systems (Cheewatrakoolpong et al., 2005). It has been recognized as the most potent activator of the NLRP3 inflammasome, which initiate an inflammatory response by releasing proinflammatory cytokines and inducing pyroptotic cell death (Pelegrin, 2021). The expression of P2RX7 has been found to be upregulated in human carotid atherosclerotic plaques, and this elevation has been correlated to the degree of coronary artery stenosis (Shi et al., 2021; Shokoples et al., 2021). These findings provide compelling evidence for the involvement of P2RX7 in the pathogenesis of atherosclerosis. P2RX7 has been shown to activate the NLRP3 inflammasome, which is required for atherogenesis, providing a potential explanation for the involvement of P2RX7 in this disease (Duewell et al., 2010; Peng et al., 2015). In addition, P2RX7 knockout mice exhibit smaller atherosclerotic plaques than wild-type mice, further highlighting the potential therapeutic value of P2RX7-targeting strategies for preventing atherosclerosis progression (Stachon et al., 2017). P2RX7 expression has been found to be increased in the substantia nigra of PD patients (Durrenberger et al., 2012). In a 6-hydroxydopamine-induced PD rat model, increased microglial activation and P2RX7 expression were found in the damaged striatum and substantia nigra (Carmo et al., 2014; Oliveira-Giacomelli et al., 2019). Treatment with Brilliant Blue G, a P2RX7 antagonist, reduced parkinsonism symptoms and prevented dopaminergic neuron death (Oliveira-Giacomelli et al., 2019). P2RX7 expression is primarily found in microglial cells rather than astrocytes or neurons, and the neuroregeneration observed in the presence of P2RX7 receptor antagonists is most likely mediated by the inhibition of microglial activation (Marcellino et al., 2010; Crabbé et al., 2019). Furthermore, α-synuclein, the key protein implicated in PD pathogenesis, can bind to P2RX7 receptors in microglia, leading to activation of microglial BV2 cell line and ultimately resulting in neuroblastoma SH-SY5Y cell apoptosis (Jiang et al., 2015). Furthermore, recent studies have demonstrated that inhibiting the assembly of the NLRP3 inflammasome in both familial and sporadic PD models leads to a reduction in dopaminergic neurodegeneration (Panicker et al., 2022). Taken together, accumulated evidences have suggested that the P2RX7/NLRP3 signaling pathway may play a crucial role in the pathogenesis of both atherosclerosis and PD. Therefore, further research should be considered to explore the potential of this pathway as a common key therapeutic target for both conditions.

These hub genes were shown to closely associated with pertussis. Several epidemiological studies have suggested that pertussis may increase the risk of PD (de Pedro-Cuesta et al., 1996; Vlajinac et al., 2013). Further research has indicated that there is no direct relationship between PD and pertussis vaccination or immunoglobulin against pertussis, suggesting that the association is not simply a result of immunological responses to the bacteria (Fiszer et al., 2004; Williams et al., 2023). On the other hand, studies on atherosclerosis have shown that oxLDL can inhibit the endothelium-dependent relaxation of arteries via pertussis toxin-sensitive G proteins (Shimokawa et al., 1991; Jing et al., 1999). Furthermore, dysfunction of endothelium-dependent relaxation has also been observed in PD patients (Yoon et al., 2015). Therefore, further research could be conducted to investigate the possibility of pertussis toxin-sensitive G protein-related endothelial dysfunction as a common pathogenic mechanism in both atherosclerosis and PD.

Cerebrovascular pathology is a prevalent finding among PD patients (Hong et al., 2018). Apart from the motor symptoms, research has highlighted the contribution of carotid atherosclerosis to microvascular damage, leading to aggravated cognitive dysfunction in PD (Kim et al., 2012, 2014). Currently, no therapy has demonstrated the ability to slow down or halt the progression of PD. However, with ongoing investigations into genetic causes and mechanisms of neuronal death, several promising strategies are under evaluation for their disease-modifying potential (Bloem et al., 2021; Vijiaratnam et al., 2021). The exploration of shared genetic factors between carotid atherosclerosis and PD could unveil vital connections and pathways, offering promising avenues for the development of treatments that may decelerate the progression of motor symptoms and cognitive dysfunction, thereby significantly benefiting PD patients. Furthermore, intriguingly, studies have indicated that long-term statin use may reduce the risk of PD (Yan et al., 2019). Notably, besides their lipid-lowering effects, statins have been shown to exert regulatory effects on the NLRP3 inflammasome, leading to anti-inflammatory effects (Koushki et al., 2021).

Previous studies have investigated critical genes associated with atherosclerosis and PD independently. However, there have been limited bioinformatic approaches to explore their shared molecular mechanisms. In this study, we identified common DEGs and hub genes in both diseases, providing insights into their pathogenesis. Nevertheless, our study has some limitations. Firstly, external validation is needed to confirm our findings. Secondly, the pathogenetic mechanism associated with these hub genes and potential therapeutic strategies requires further validation through *in vitro* and *in vivo* experiments, which will be the focus of our future research.

In conclusion, we identified common DEGs between atherosclerosis and PD and conducted enrichment and PPI network analysis. Our results suggest that these diseases may share a pathogenic mechanism involving several immune-related systems, including the complement system, the BAFF/BAFF-R signaling pathway, and the P2RX7/NLRP3 signaling pathway. This study provides a foundation for future research on the molecular mechanisms underlying atherosclerosis and PD.

## Data availability statement

Publicly available datasets were analyzed in this study. This data can be found here: GSE7621, GSE49036, GSE28829, and GSE100927.

## Author contributions

QW contributed to data analyses, as well as writing and revising the manuscript. QX revised the manuscript. Both authors read and approved the final manuscript, contributed to the article, and approved the submitted version.

## Footnotes

1
http://www.ncbi.nlm.nih.gov/geo/

2
https://string-db.org/

## Supplementary material

Supplementary Figure 1 Differentially expressed genes (DEGs) analysis of individual dataset: hierarchical clustering heatmap **(A)**, PCA plot **(B)**, and volcano plot **(C)** of GSE100927; hierarchical clustering heatmap **(A)**, PCA plot **(B)**, and volcano plot **(C)** of GSE7621.

Supplementary Figure 2 Venn diagrams of common DEGs and infiltrating immune cells: **(A)** Venn diagrams of common DEGs identified by atherosclerosis dataset GSE28829 and PD dataset GSE49036; **(B)** Venn diagrams of common infiltrating immune cells identified by atherosclerosis dataset GSE28829 and PD dataset GSE49036.

Supplementary Figure 3 Validating the diagnostic efficacy of hub genes: Wilcoxon test **(A)** and ROC curve **(B)** of the diagnostic efficacy of hub genes in atherosclerosis dataset GSE100927; Wilcoxon test **(C)** and ROC curve **(D)** of the diagnostic efficacy of hub genes in PD dataset GSE7621.
