# Supplementary figures and images for: Bioinformatics analysis of potential common pathogenic mechanism for carotid atherosclerosis and Parkinson’s disease
Source: Front Aging Neurosci. 2023 Aug 15;15:1202952. doi: 10.3389/fnagi.2023.1202952 (PMC10464527; doi:10.3389/fnagi.2023.1202952)

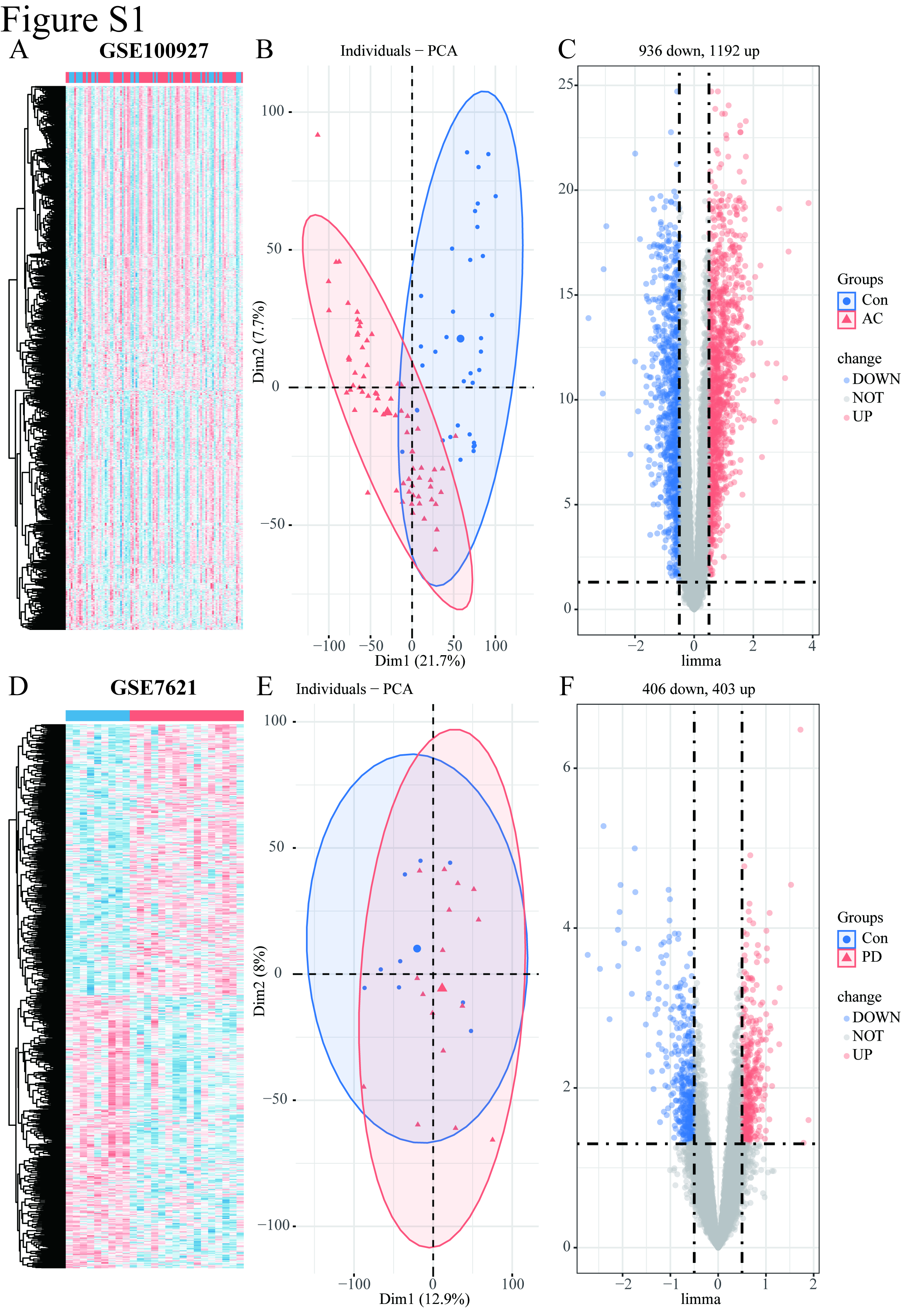

Supplement: Supplementary Figure 1 — Differentially expressed genes (DEGs) analysis of individual dataset: hierarchical clustering heatmap (A), PCA plot (B), and volcano plot (C) of GSE100927; hierarchical clustering heatmap (A), PCA plot (B), and volcano plot (C) of GSE7621. [file Image_1.TIF]

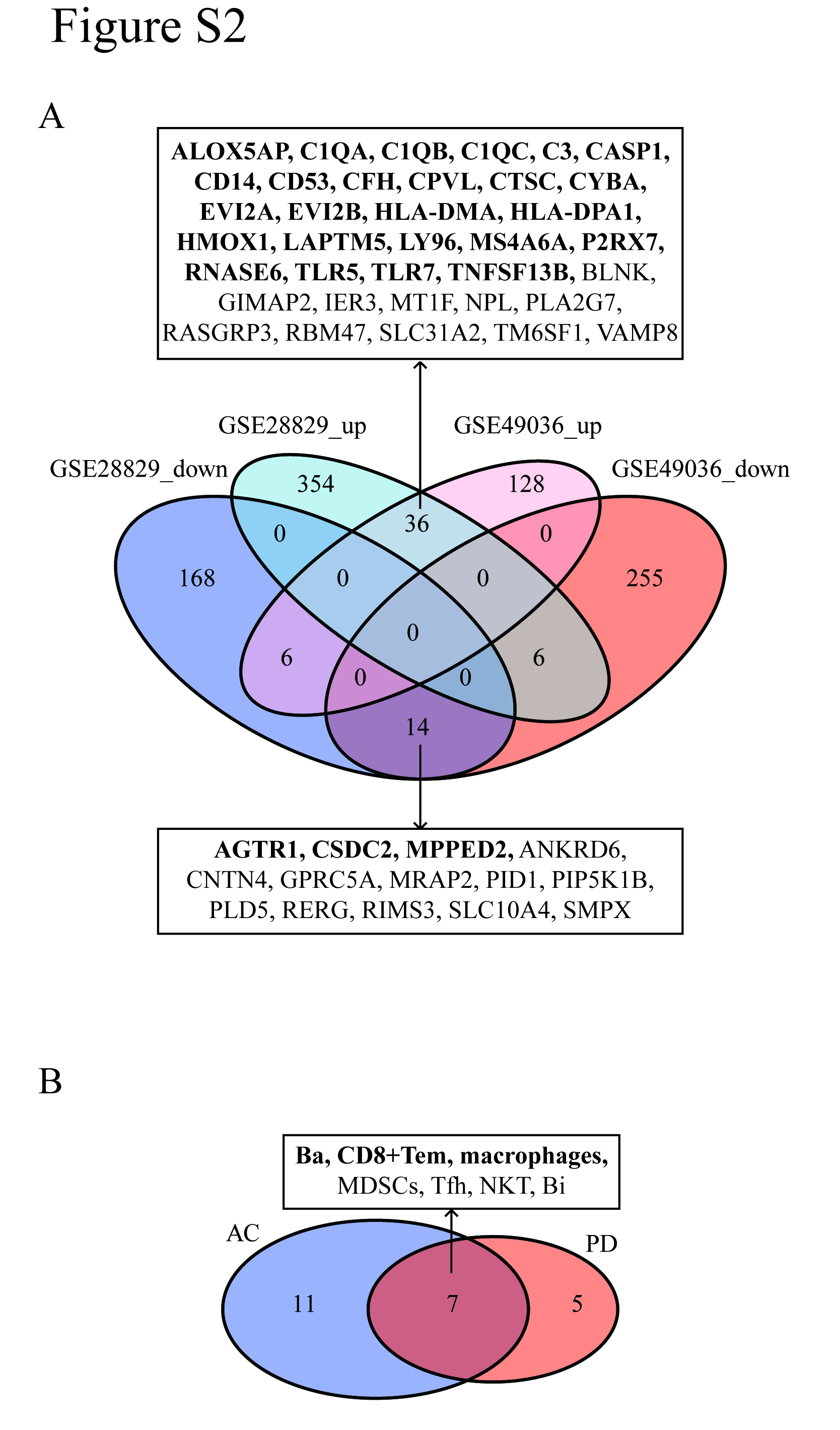

Supplement: Supplementary Figure 2 — Venn diagrams of common DEGs and infiltrating immune cells: (A) Venn diagrams of common DEGs identified by atherosclerosis dataset GSE28829 and PD dataset GSE49036; (B) Venn diagrams of common infiltrating immune cells identified by atherosclerosis dataset GSE28829 and PD dataset GSE49036. [file Image_2.TIF]

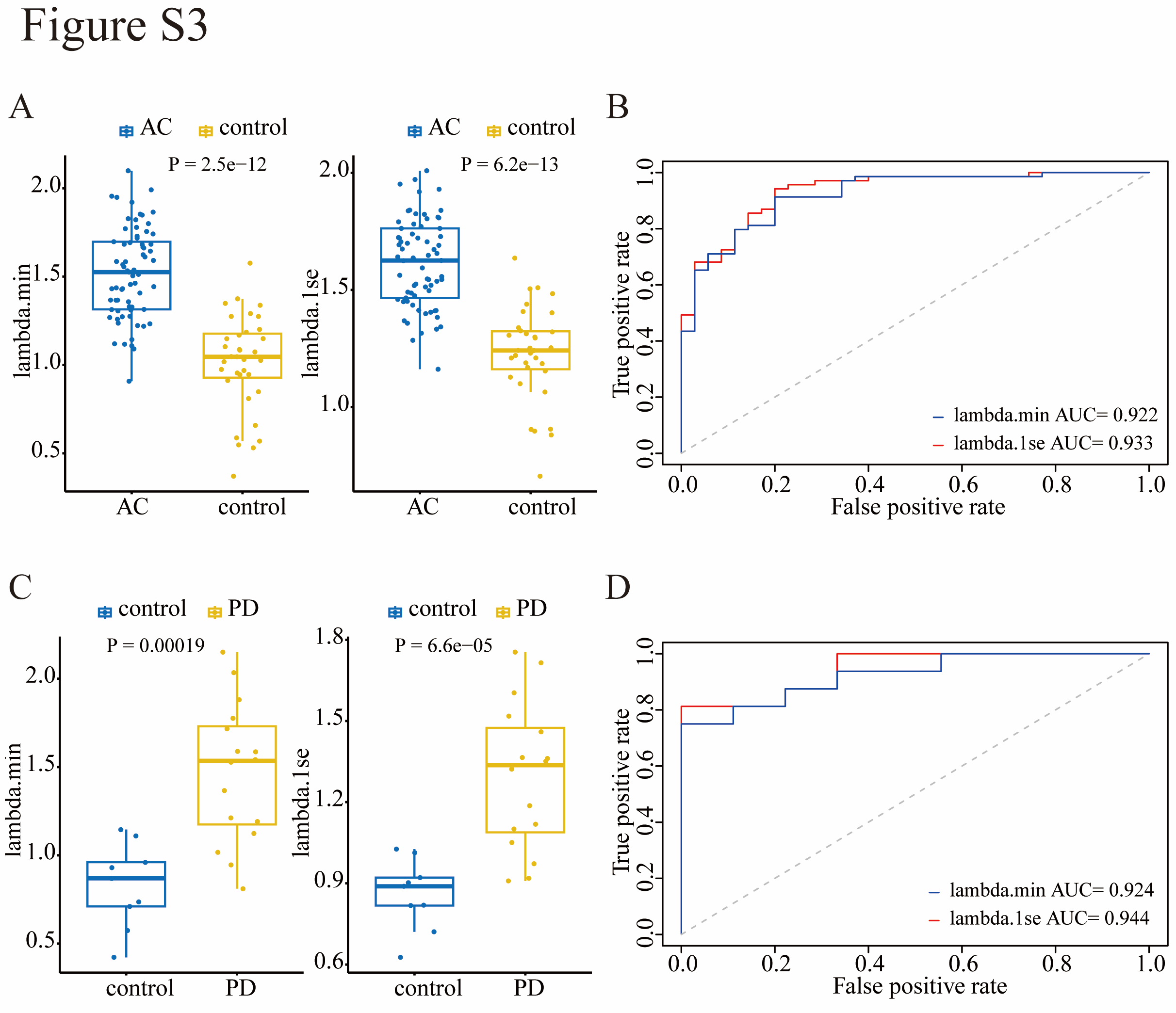

Supplement: Supplementary Figure 3 — Validating the diagnostic efficacy of hub genes: Wilcoxon test (A) and ROC curve (B) of the diagnostic efficacy of hub genes in atherosclerosis dataset GSE100927; Wilcoxon test (C) and ROC curve (D) of the diagnostic efficacy of hub genes in PD dataset GSE7621. [file Image_3.TIF]
